# Relationship between Dietary Fiber Fermentation and Volatile Fatty Acids’ Concentration in Growing Pigs

**DOI:** 10.3390/ani10020263

**Published:** 2020-02-07

**Authors:** Jinbiao Zhao, Yu Bai, Gang Zhang, Ling Liu, Changhua Lai

**Affiliations:** State Key Laboratory of Animal Nutrition, Ministry of Agriculture and Rural Affairs Feed Industry Centre, China Agricultural University, Beijing 100193, China

**Keywords:** correlation, digestibility, dietary fiber, pig, short chain fatty acid

## Abstract

**Simple Summary:**

The study suggests differences in fermentable capacity of fibrous feed ingredients are associated with fiber composition in pig. Results demonstrate that the fiber digestibility of oat bran, sugar beet pulp and soybean hulls is greater than for corn bran, wheat bran and rice bran in the pig intestine. Furthermore, results indicate that volatile fatty acids’ concentration (VFA) is positively correlated primarily with insoluble dietary fiber (IDF) fermentation, and the digestibility of IDF is the best single variable to predict fecal VFA concentrations. The contribution of this study is to provide instructions on how to implement fiber-rich ingredients effectively in the feed formulation for swine.

**Abstract:**

This study was conducted to determine whether differences in fiber fermentation in fiber-rich feed ingredients exist and to assess relationship between fiber fermentation and concentration of volatile fatty acids (VFA) in pig. Castrated males (barrows) were allotted randomly to six diets formulated with different amounts of wheat bran (WB), corn bran (CB), sugar beet pulp (SBP), oat bran (OB), soybean hulls (SH) or rice bran (RB). The apparent ileal digestibility (AID) of soluble dietary fiber (SDF) for OB and SH diets was greater (*P* < 0.05) than for the other diets. The fermentation of total dietary fiber (TDF) and insoluble dietary fiber (IDF) in the hindgut were greater (*P* < 0.05) for SBP and SH diets than for WB, CB, OB and RB diets. The apparent total tract digestibility (ATTD) values of all fiber components in SBP, SH and OB diets were greater (*P* < 0.05) than for WB, CB and RB diets. The concentration of VFA in feces was positively correlated with the ATTD of IDF and cellulose, and ATTD of IDF is the best factor for predicting fecal VFA concentration. Overall, dietary fiber source affected fermentable characteristics of fiber components in the different digestive segments of pig intestine.

## 1. Introduction

Dietary fiber is resistant to digestion by endogenous enzymes in the small intestines of pigs, but can be partially or completely fermented by microbiota in the hindgut to produce volatile fatty acids (VFA) that are absorbed to provide energy [1]. The production of VFA by fiber fermentation plays an important role in regulating host metabolism, immune system and cell proliferation [2,3]. It can not only bind to the G protein-coupled receptors (GPRs) that are expressed on both intestinal epithelia and immune cells [4], but also modulates satiety and gut motility via activation of GPR43 on enteroendocrine L-cells to stimulate the release of the anorectic gut hormones, such as glucagon-like peptide-1 and peptide YY [5]. Dietary fiber is mainly composed of soluble dietary fiber (SDF) and insoluble dietary fiber (IDF), and SDF is easier to ferment by gut bacteria compared to IDF [6,7]. Soluble fibers include pectin, gum, β-glucan and hemicellulose, whereas cellulose consists of insoluble fibers [8]. Traditionally, dietary fiber is thought to be fermented in the hindgut of the pig, but recent studies reported that it is also fermented in the small intestine, since fiber-degrading bacteria are found in the upper gut of the pig [9,10]. However, there is large amount of variation in the fermentation potential of fiber components from highly fermentable pectin to cellulose that is slowly fermentable. Thus, considerations of fiber fractions fermentation in feedstuffs and estimating their nutritional values are critically important for the swine industry.

In our previous study, we reported that sources of dietary fiber, derived from wheat bran (WB), corn bran (CB), sugar beet pulp (SBP), oat bran (OB), soybean hulls (SH) and rice bran (RB), affected VFA production and composition of gut microbiota in the intestine of the pig due to their differences in chemical composition and physical properties, and VFA produced by gut microbiota to ferment dietary fiber is positively correlated to IDF content in fiber-rich ingredients, not SDF [11]. However, the relationship between VFA production and fermentable capacity of various fiber components is not known, and no previous studies have been focused on the development of predictive equations for VFA concentration. Based on our previous study, we put forward the hypothesis that different sources of dietary fiber among fibrous feed ingredients vary in degree of fermentation in the different segments of the digestive tract of pigs, and VFA production is positively correlated to the fermentable capacity of insoluble fibers.

Therefore, the objectives of this study were to determine the effects of dietary fiber sources from feed ingredients with equally total dietary fiber (TDF) content on nutrient digestibility and fiber fermentation in different segments of the intestinal tracts of pigs and to determine the relationship between the fermentation of different dietary fiber components and concentration of VFA in the ileal digesta and feces of the pig.

## 2. Materials and Methods

The Laboratory Animal Welfare and Animal Experimental Ethical Inspection Committee in China Agricultural University reviewed and approved all protocols used in this study (number AW12119102-2-1). The animal trial was conducted at the Swine Research Unit of China Agricultural University (Beijing, China).

### 2.1. Animals and Housing

Thirty-six Duroc × (Landrace × Yorkshire) crossbred barrows (initial body weight of 48.5 ± 2.1 kg) were fitted surgically with a T-cannula in the distal ileum, as described by Stein et al. [12]. Pigs were allowed a 15-day recovery period after surgery, and a corn-soybean meal diet was fed during this period. The composition of ingredients and the analyzed nutritive levels in the corn-soybean meal diet are presented in Supplementary Table S1. All pigs were housed in individual stainless-steel metabolism crates (1.4 × 0.7 × 0.6 m) equipped with nipple drinking devices and feed boxes. The room temperature was maintained at 20–25 °C throughout the experiment.

### 2.2. Experimental Design and Diets

The pigs were allotted randomly to 6 dietary treatments, for a total of 6 pigs per treatment. The different dietary treatments were formulated with either WB, CB, SBP, OB, SH or RB as the sole dietary fiber source for each dietary treatment. The chemical compositions of WB, CB, SBP, OB, SH and RB were analyzed. All high-fiber diets were formulated to contain the same level (15%) of TDF by adjusting the inclusion level of WB, CB, SBP, OB, SH and RB in each diet. The feed ingredients and chemical compositions of diets were analyzed, and results are summarized in Table 1. Vitamins and minerals were included in all diets to meet or exceed the nutrient requirements for growing pigs [13]. All diets contained 0.3% chromic oxide as an indigestible marker. The experiment lasted 21 d, including 15 d for diet adaptation, 3 d for fecal collection and 3 d for digesta collection.

Pigs were provided water ad libitum and fed a daily amount of feed equivalent to 4% of body weight divided equally into two meals provided at 08:00 and 16:00. Body weights of individual pigs were recorded at the beginning of the experiment to calculate the feed allowance.

### 2.3. Sample Collection

Fecal samples were collected from 08:00 to 18:00 from day 16 to day 18 of the experiment via grab-sampling. The pallet was placed at the bottom of each cage to prevent contamination of fecal samples. During the 3 d collection period, feces were immediately collected into plastic bags and stored at −20 °C. Digesta samples were collected from day 19 to day 21 of the experiment at 08:00 and the last sample was obtained at 18:00 on each collection day. For digesta collection, a plastic bag was attached to the barrel of the cannula using a cable tie. Bags were removed when filled and stored at −20 °C to prevent bacterial degradation of the samples. At the end of this animal trial, digesta and fecal samples from the same pig were thawed and mixed. Sub-samples of digesta (about 300 g) and feces (about 200 g) were lyophilized and then ground through a 1 mm screen. All sub-samples of ileal digesta and feces were stored at −20 °C prior to chemical analysis.

### 2.4. Chemical Analyses

The dry matter (DM) (Method 934.01), starch (Method 979.10), crude protein (Method 990.03), ether extract (EE) (Method 920.39), chromium (Method 990.08), ash (Method 942.05), SDF and IDF (Method 991.43; Ankom ^TDF^ Analyzer) were analyzed according to the procedures of the Association of Official Analytical Chemists [14]. Organic matter (OM) was calculated by difference between DM and ash. Total dietary fiber (TDF) was calculated as the sum of SDF and IDF. Neutral detergent fiber (NDF) and acid detergent fiber (ADF) were determined using fiber bags (Model F57, Ankom Technology, Macedon, NY, USA) and a fiber analyzer (ANKOM^200^ Fiber Analyzer, Ankom Technology, Macedon, NY, USA) after adaptation of the procedure described by Van Soest et al. [15]. The concentration of aNDF was analyzed using heat stable α-amylase and sodium sulphite without correction for insoluble ash. The content of acid detergent lignin (ADL) was also determined according to the method of Ankom Technology. Hemicellulose was calculated as the difference between aNDF and ADF, and cellulose was calculated as the difference between ADF and ADL. Non-starch polysaccharide (NSP) was the difference as TDF and ADL. All samples of ingredients and diets were analyzed for water binding capacity [16]. Values for water binding capacity were expressed as the amount of water retained by the pellet (g/g). The gross energy (GE) of feces, diets and ingredients were determined using an Automatic Isoperibol Oxygen Bomb Calorimeter (Parr 1281 Calorimeter, Moline, IL, USA). The analysis and concentration of VFA in the ileal digesta and feces were cited from our previous publication [11]. About 1.0 g samples of ileal digesta and feces were put into 10 mL centrifuge tubes with 2.0 mL 0.10% hydrochloric acid, and incubated on ice for 25 min. The tubes were mixed and centrifuged at 15,000 rpm for 15 min. The supernatant was filtered using a 0.20 mm Nylon Membrane Filter (Millipore, Bedford, OH, USA) and poured into a Gas Chromatograph System (Agilent HP 6890 Series, Santa Clara, CA, USA) for VFA determination.

### 2.5. Calculations

The apparent ileal (AID) and total tract digestibility (ATTD) of DM, OM, GE, EE, ADF, NDF, cellulose, hemicellulose, TDF, SDF and IDF in ileal digesta or feces were calculated for all diets [17].

AD = 1 − (CN_digesta or feces_/CN_diet_) × (Cr_diet_/Cr_digesta or feces_).

In this equation, AD is the AID or ATTD of energy or nutrients and fiber components fermentation of ileal digesta or feces in a diet (%); CN_digesta or feces_ is the energy and nutritive level of ileal digesta or feces; CN_diet_ is the energy and nutritive level of a diet.

The digestible energy (DE, MJ/kg) in ileal digesta and feces of pigs was calculated according to the following equation:

DE = GE_diet_ × AD_energy_

where GE_diet_ is GE content in each diet, and AD_energy_ is the AID or ATTD of GE in each diet.

The disappearance (%) of DM, GE, EE, ADF, NDF, OM cellulose, hemicellulose, TDF, SDF and IDF and the content of DE (MJ/kg) in the hindgut were calculated according to the following equation:

Hindgut disappearance = ATTD − AID

DE_hindgut_ = GE_diet_ × ATTD_energy_ − GE_diet_ × AID_energy_

### 2.6. Statistical Analysis

All the data were checked for normality and outliers were detected using the UNIVARIATE procedure of SAS (SAS Inst. Inc., Cary, NC, USA). No outliers were found. All data were analyzed using the MIXED procedure of SAS 9.2 with individual pig as the experimental unit. The statistical model included the fixed effect of diet and the random effects of individuals pig. Statistical differences among the treatments were separated by Tukey’s multiple range test. Treatment means were calculated using the LSMEANS statement. Difference was considered significant at *P* < 0.05. Pearson’ s coefficient correlations between dietary fiber digestibility in the ileum and feces of the pig and VFA concentration were analyzed using the PROC CORR procedure of SAS 9.2. Prediction equations for acetate, propionate, butyrate and total VFA concentrations in the feces of pigs fed 6 dietary treatments were developed using PROC REG of SAS. Stepwise regression was used to determine effects of ATTD of different fiber components on fecal VFA concentrations. Variables with *P* < 0.10 were retained in the model and the R^2^ values were used to define the best-fit models.

## 3. Results

There was no residual feed left by any pig during the entire experiment. All the pigs were healthy with no clinical symptoms or cannula losses.

### 3.1. The Chemical Compositions and Physical Characteristics of Ingredients

Wheat bran, CB, SH and RB have a low SDF/IDF ratio, while OB and SBP have a high content of SDF content, SDF/IDF ratio and water binding capacity (Table 2). The RB contains the lowest contents of TDF and NSP compared with the other feed ingredients. In addition, CB and OB have more hemicellulose than WB, SBP, RB and SH, but the contents of ADL in SBP and RB were greater than those in WB, CB, SH and OB.

### 3.2. The Apparent Ileal Digestibility (AID) of Chemical Components

The AID of GE and DE content in the OB diet were greater (*P* < 0.05) than for CB, SBP, SH and RB diets (Table 3). Pigs fed WB and OB diets had greater (*P* < 0.05) AID of DM and OM than pigs fed the other dietary treatments. The AID values of ash and EE in the SBP diet were the lowest (*P* < 0.05) among all diets. Pigs fed the OB diet had greater (*P* < 0.05) AID of TDF than those fed the WB, CB, SBP, SH and RB diets. The AID of NDF and hemicellulose in the OB diet were greatest (*P* < 0.05) compared with the other dietary treatments.

### 3.3. The Apparent Total Tract Digestibility (ATTD) of Chemical Components

The DE contents and the ATTD of GE and OM in SBP and OB diets were greater (*P* < 0.05) compared with the WB, CB, SH and RB diets (Table 4). Pigs fed WB and RB diets had less (*P* < 0.05) ATTD of ash than pigs fed the CB, SBP, OB and SH diets. The ATTD values of EE in pigs fed the SBP diet were the lowest (*P* < 0.05) among all dietary treatments. Pigs fed SBP, OB and SH diets had greater (*P* < 0.05) ATTDs of TDF, SDF, IDF, NDF, ADF, cellulose and hemicellulose compared with pigs fed WB, CB and RB diets.

### 3.4. The Hindgut Disappearance of Chemical Components

The disappearance of GE, DE, OM, DM, ADF and cellulose in the hindguts of pigs fed SBP and SH diets were greater (*P* < 0.05) than in pigs fed the WB, CB, OB and RB diets (Table 5). The hindgut disappearance of ash was less (*P* < 0.05) for pigs fed the RB diet compared to values for pigs fed the other diets. Pigs fed SBP, OB and SH diets had greater (*P* < 0.05) fermentation of TDF and NDF in the hindgut than pigs fed WB, CB and RB diets. The fermentation of SDF in the hindguts of pigs fed the SBP diet was greatest (*P* < 0.05) among all dietary treatments.

### 3.5. Correlations between Fiber Components’ Digestibility and Concentrations of VFA in the Ileal Digesta and Feces

In the samples of ileal digesta (Table 6), the AID of GE was positively correlated with the AID of DM and OM (*P* < 0.05), and the total VFA concentration showed a significant correlation with the AID of cellulose (*P* < 0.05). In the fecal samples, concentration of acetate was positively correlated with the ATTD of IDF (*P* < 0.05) (Table 7). The ATTD of GE was positively correlated (*P* < 0.05) with the ATTD of DM, OM, NDF, ADF, hemicellulose and TDF. There were positive correlations among the ATTD values of different fiber fractions, such as TDF, SDF, IDF, NDF, ADF, cellulose and hemicellulose. Prediction equations for acetate, propionate, butyrate and total VFA concentrations in the feces of pigs fed six dietary treatments were developed (Table 8). No suitable predictive equation for fecal propionate concentration was observed using the ATTD of different fiber components (*P* > 0.10). The predictive equation for fecal acetate concentration using a combination of ADF and SDF digestibility (*R*^2^ = 0.85; *P* = 0.06) is better than those equations using the ATTD of ADF (*R*^2^ = 0.55; *P* = 0.09) or IDF (*R*^2^ = 0.72; *P* = 0.03). The predictive equations for fecal butyrate (*R*^2^ = 0.65; *P* = 0.05) and total VFA concentrations (*R*^2^ = 0.61; *P* = 0.07) were developed by using the ATTD of IDF.

## 4. Discussion

In the present study, results indicated that the apparent ileal digestibility (AID) of dietary fiber fractions ranged from 0.70% (the AID of cellulose) to 48.93% (the AID of SDF). Those results support those reported by Bach Knudsen et al. [18], who found that the apparent ileal digestibility (AID) of NSP, which is the difference between TDF and lignin, in pigs, ranged from −7% to 40%. Importantly, some studies have reported that both the gastrointestinal digesta and the feces contain a significant amount of endogenous fibrous components from nondietary materials that interfere with the determination of dietary fiber fermentation [19,20]. The fermentation capacity of dietary fiber that is not corrected for the endogenous loss of fiber components can greatly underestimate dietary fiber fermentation. Therefore, there was a limitation to evaluating the fermentation of high-fiber feed ingredients in our study owing to neglecting the endogenous loss of fiber components. The apparent total tract digestibility (ATTD) of SDF in all dietary treatments, except for RB diet, in the present study, was greater than IDF, which supports results of Urriola and Stein [21], indicating an average apparent total tract digestibility (ATTD) of SDF of 20% units greater than IDF. A small difference in the apparent total tract digestibility (ATTD) of SDF and IDF in the RB diet may be associated with the high degree of polysaccharide polymerization of RB, resulting in depressed SDF digestibility. The high DE content in the ileum, and apparent ileal digestibility (AID) of GE, TDF, NDF and cellulose in the OB diet, were associated with the high contents of SDF and β-glucan compared with other diets. That indicates that fiber fermentation for the OB diet provided more energy in the small intestine compared with the other dietary treatments. That result is consistent with a previous report indicating that β-glucan is highly fermentable in the small intestine because of its solubility characteristics compared with those of xylose and arabinose [22]. In spite of a high content of SDF in SBP, the lower apparent ileal digestibility (AID) of GE, SDF, NDF and hemicellulose may result from the high portion of ADL, which is hardly fermented by bacteria in the intestines of pigs [23]. The greatest ileal fermentation of SDF in the SH diet was associated with its physicochemical properties and the high contents of fermentable oligosaccharides and soluble NSP. The greater apparent ileal digestibility (AID) of DM and OM in the WB diet resulted from its structure and physical properties, such as bulking, compared with that for CB, SBP, SH and RB diets.

In the present study, pigs fed the OB, SBP and SH diets had greater apparent total tract digestibility (ATTD) of all fiber fractions compared with values for pigs fed CB, WB and RB diets, resulting in a higher DE content in the total digestive tract. The higher fermentable capacity of OB, SBP and SH was mainly associated with higher SDF content, leading to more retention time of fiber fractions in the intestines of pigs. Chabeauti et al. [24] found that the apparent total tract digestibility (ATTD) of NSP in growing pigs varies from 16.3% for wheat straw, 43.5% for WB and 69.5% for SBP to 79.1% for SH. The poor fiber fermentation of WB is ascribed to its high insoluble fiber content that makes the dietary fiber less fermentable compared with highly fermentable pectin substances in SBP and SH [25]. The higher apparent total tract digestibility (ATTD) of GE for the pigs fed SBP, OB and SH diets was due to the greater capacity for fermentation of fiber fractions than for WB, CB and RB diets. Those results are consistent with the findings of Urriola and Stein [21], which showed that pigs fed the 30% SBP or SH diets had greater apparent total tract digestibility (ATTD) of GE and TDF compared with pigs fed a 30% corn distillers dried grains with soluble (DDGS) diet. Lyu et al. [26] also reported that the apparent total tract digestibility (ATTD) of GE, DM and NDF in the diet with 30% OB was greater than for a diet with 30% WB fed to growing pigs. Pigs fed the SBP and SH diets had greater fermentation of GE, DM, OM, TDF, IDF, NDF, ADF and cellulose in their hindguts than those pigs fed the WB, CB and RB diets. Those results indicated that fermentation of fibrous fractions and the digestibility of non-fibrous components in the gut of pigs were improved due to the existence of more fermentable SDF, resulting from the longer retention time of digesta. The lower fermentation of GE, DM, OM, SDF, ADF and cellulose in the hindguts of pigs fed the OB diet was due to less of the substrates entering into large intestine, resulting from the high fermentation capacity in the foregut of the pig. The greater hindgut disappearance of SDF in the SBP diet may be attributed to more SDF fractions of SBP flowing into the hindguts of pigs as substrates because of the low ileal fermentation of fiber. The fermentation of hemicellulose in the hindgut was not different among dietary treatments which indicated that most of the hemicellulose was fermented in the small intestines of pigs. Jaworski and Stein [27] reported that the ileal fermentation of insoluble hemicellulose in some high-fiber diets and ingredients was close to the apparent cecal digestibility of insoluble hemicellulose, indicating that degradation of these fractions is negligible in the cecum. The results showed the apparent total tract digestibility (ATTD) of EE in the feces was lower than the apparent ileal digestibility (AID) of EE in the ileal digesta of pigs. The negative values for the fermentation of EE in the hindgut in the present study are consistent with previous reports [21,28]. The negative values are most likely a consequence of microbes in the hindgut using carbohydrates for synthesis of fatty acids, as well as endogenous VFA from non-dietary components.

It is accepted that the concentration of VFA in the ileal digesta and feces of pig accounts for a small proportion of VFA production [29], because most VFA produced by gut bacteria was absorbed. Meanwhile, the endogenous mucin and microbial cells in the pig intestine are rich in IDF fractions, resulting in the production of VFA derived from non-dietary components’ fermentation, as well as dietary fiber fermentation [20]. It was also a limitation that the role of VFA produced by fermenting non-dietary components was neglected in our study. In our previous study [11], the high lactate content in the ileal digesta of pigs fed the OB diet is consistent with the current result for more AID of TDF compared to other dietary treatments, which indicates that fiber fermentation of OB primarily produced more lactate in the small intestines of pigs than other fiber fractions from CB, WB, SBP, SH and RB. Bach Knudsen and Canibe reported high concentrations and flow of lactate in the ilea of cannulated pigs after feeding them a diet supplemented with OB because of its high content of β-glycan [30]. The concentration of VFA in the feces of pigs fed the SH diet was the greatest compared with all other diets, resulting from its greater content of fermentable oligosaccharides and soluble NSP, similar to results of Freire et al. [31]. Fiber sources affected total VFA concentration in the ileal digesta and feces of pigs in our previous study [11], in which the pigs fed the SBP, OB and SBP had higher VFA concentrations compared to those fed the WB, CB and RB diets. However, it has been reported that there is no difference in total VFA in the ileal digesta of pigs fed diets with WB, SBP, corn DDGS, pea hulls or pea inner fiber [9]. Those inconsistent results for differences in VFA concentration among various feed ingredients were associated with dietary fiber level and pig body weight. Carneiro et al. [32] compared the effects of two fiber sources, WB and maize cobs in pigs and found no differences in the amounts of VFA in the small intestine; however, the pigs fed the maize cobs diet showed the greater proportion of acetate and a lower proportion of butyrate, which accounted for the total VFA in the cecum, compared to those fed the WB diet. Therefore, the variation in fermentability among different dietary fibers is not only associated with the amount of substrate, but also the source of the fiber fraction and its physical characteristics. It is worth mentioning that a high concentration of copper (100 mg/kg vs. 4 mg/kg) was provided to the pigs in the present study to be close to actual swine production in China and maintain a good performance of pigs in comparison to recommendation requirement of NRC (2012). The antimicrobial potentials of copper and zinc have been reported in many previous studies [33,34]. However, a publication indicated the diets with 150 mg/kg of copper from copper hydroxychloride had no significantly negative effects on the concentrations of VFA in growing pigs, although the activity of gut microbiota may be impaired by a high concentration of copper [35]. Overall, the effects of trace minerals and antibiotic supplementation on fiber fermentation and VFA production should be clarified in further study.

In the present study, concentrations of acetate, propionate, butyrate and total VFA were positively correlated with the apparent total tract digestibility (ATTD) values of insoluble fiber fractions, which agrees with results from numerous studies in which there was a positive relationship between fermentation of insoluble fiber sources and concentration of butyrate in the large intestine [36,37]. The development of prediction equations for fecal VFA concentrations indicated that the digestibility of IDF is the best single variable with which to predict the concentration of VFA in the feces of pigs compared to the apparent total tract digestibility (ATTD) of other fiber components. However, the prediction equations for fecal VFA were developed on a basis of six dietary treatments in the present study, which were formulated with six different fiber-rich ingredients. The fermentable capacities of more fiber-rich feeds should be added into the prediction equations for VFA concentration to improve the accuracy and precision of the equations. In addition, some studies found that soluble fractions of dietary fiber are fermented faster and yield greater amounts of VFA than for fermentation of insoluble fractions [6,38]. Those different results indicated that concentration of VFA is not only associated with the chemical composition of fiber, but also their physical characteristics and molecular structure. On the other hand, in spite of the higher fermentable capacity of SDF, the feed ingredients, such as SBP and OB, are rich in SDF, but they are not always used to feed animals owing to their supplies and prices. In contrast, insoluble fibrous feed ingredients are common in the formulations for swine. Therefore, considering the lower fermentable capacity of IDF and the positive relationship between IDF digestibility and VFA concentrations, it is necessary that nutritionists continue to explore ways to improve insoluble fiber fermentation and increase the energy supplied, such as through feed processing technology and enzyme supplementation. It is also crucial to explore the interactions of fiber fermentation between fiber sources and levels in the formulation of pig diets to quantify the requirement of dietary fiber, resulting in the achievement of efficient utilization of fibrous ingredients.

## 5. Conclusions

The fermentable capacity of fiber components derived from various fiber-rich ingredients varies in the different segments of the digestive tracts of pigs owing to their different chemical compositions and physical properties. The high concentrations of VFA in feces of pigs were associated with the fermentation of IDF. Those differences in the fermentable capacity of fiber among different fibrous ingredients provide a reference for the use of various high-fiber ingredients in formulated diets for pigs, in accordance with the positive correlation between VFA concentration and IDF digestibility. The ways to improve IDF fermentation should be considered to increase the energy supplied and the prebiotic effects of VFA for pigs, improving the sustainability of the swine production.

## Figures and Tables

**Table 1:** The composition of experimental diets ^1^.

	Diets					
Items	WB	CB	SBP	OB	SH	RB
Ingredients, g/kg						
Corn starch	350.8	453.6	459.5	346.3	451.3	214.3
Soy protein isolate	120.0	120.0	140.0	120.0	140.0	110.0
RB	-	-	-	-	-	500.0
WB	357.0	-	-	-	-	-
CB	-	250.0	-	-	-	-
SBP	-	-	225.0	-	-	-
OB	-	-	-	358.0	-	-
SH	-	-	-	-	235.0	-
Soy oil	30.0	30.0	30.0	30.0	30.0	30.0
Sucrose	100.0	100.0	100.0	100.0	100.0	100.0
Limestone	11.0	5.5	-	5.0	-	5.0
Dicalcium phosphate	8.0	6.0	22.0	18.0	21.0	18.0
*L*-Lysine-HCl	4.0	4.8	3.5	4.2	3.0	3.2
*DL*-Methionine	1.2	1.6	1.5	-	1.2	1.2
*L*-Threonine	1.5	2.0	2.0	2.0	2.0	1.8
Cr_2_O_3_	3.0	3.0	3.0	3.0	3.0	3.0
NaCl	4.5	4.5	4.5	4.5	4.5	4.5
K_2_CO_3_	3.0	3.0	3.0	3.0	3.0	3.0
MgO	1.0	1.0	1.0	1.0	1.0	1.0
Premix ^2^	5.0	5.0	5.0	5.0	5.0	50
Chemical analyses, g/kg DM basis						
GE, MJ/kg	18.23	18.47	18.18	17.99	18.30	18.06
CP	127.5	133.2	137.6	142.8	133.3	152.2
Ash	44.2	43.0	49.0	60.0	48.6	85.2
OM	955.8	957.0	951.0	940.0	951.4	914.8
EE	22.4	18.3	7.3	58.0	15.9	15.7
TDF	164.1	171.3	165.8	165.4	174.2	163.2
SDF	14.4	7.7	65.4	75.0	32.9	14.5
IDF	149.7	163.6	100.4	90.4	141.3	148.7
NDF	140.7	179.3	136.1	179.6	181.1	150.6
ADF	38.7	46.4	59.7	31.5	108.0	59.6
Cellulose	30.6	39.2	43.2	20.0	92.9	10.3
Hemicellulose	102.0	132.9	76.4	148.1	73.1	90.9

^1^ ADF = acid detergent fiber; ADL = acid detergent lignin; CB = corn bran; CP = crude protein; DM = dry matter; EE = ether extract; IDF = insoluble dietary fiber; NDF = neutral detergent fiber; OB = oat bran; OM = organic matter; RB = rice bran; SDF = soluble dietary fiber; SBP = sugar beet pulp; SH = soybean hulls; TDF = total dietary fiber; WB = wheat bran. ^2^ Premix provided the following quantities per kilogram of the complete feed for growing pigs: vitamin A, 5512 IU; vitamin D_3_, 2200 IU; vitamin E, 64 IU; vitamin K_3_, 2.2 mg; vitamin B_12_, 27.6 ug; riboflavin, 5.5 mg; pantothenic acid, 13.8 mg; niacin, 30.3 mg; choline chloride, 551 mg; Mn (MnO), 40 mg; Fe (FeSO_4_·H_2_O), 100 mg; Zn (ZnO), 100 mg; Cu (CuSO_4_·5H_2_O), 100 mg; I (KI), 0.3 mg; Se (Na_2_SeO_3_), 0.3 mg.

**Table 2:** The chemical components of different high-fiber feed ingredients (g/kg, DM basis) ^1^.

Items ^2^	WB	CB	SBP	OB	SH	RB
GE, MJ/kg	18.82	18.80	18.66	19.93	17.74	21.68
CP	193	161	113	227	221	151
NDF	372	565	435	421	679	490
ADF	107	176	245	95	488	291
ADL	22	24	69	33	19	109
Cellulose	85	152	175	62	469	182
Hemicellulose	264	389	190	326	191	199
TDF	480	686	805	494	739	331
SDF	50	76	316	194	149	57
IDF	430	609	489	299	590	274
SDF/IDF, %	11.6	12.5	64.6	64.9	25.3	20.8
NSP	458	662	735	461	720	223
Starch	167	145	76	279	95	291
Water binding capacity, g/g	5.14	5.32	9.52	7.86	5.7	3.91

^1^ Analyses were conducted in duplicate. ^2^ ADF = acid detergent fiber; ADL = acid detergent lignin; CB = corn bran; CP = crude protein; DM = dry matter; EE = ether extract; IDF = insoluble dietary fiber; NDF = neutral detergent fiber; OB = oat bran; OM = organic matter; RB = rice bran; SDF = soluble dietary fiber; SBP = sugar beet pulp; SH = soybean hulls; TDF = total dietary fiber; WB = wheat bran. OM, TDF, cellulose and hemicellulose were calculated with the following equations: OM = DM − ash; TDF = SDF + IDF; cellulose = NDF − ADF; hemicellulose = ADF − ADL; non-starch polysaccharide (NSP) = TDF − ADL.

**Table 3:** The AID values of chemical components for pigs fed diets with different high-fiber ingredients ^1^.

	Diets							
Items, % ^2^	WB	CB	SBP	OB	SH	RB	SEM	*P*-Value
DE, MJ/kg	12.52 ^b^	12.23 ^bc^	12.15 ^bc^	12.95 ^a^	12.07 ^c^	12.09 ^c^	0.10	<0.01
GE	75.43 ^ab^	72.04 ^b^	72.89 ^b^	77.77 ^a^	71.87 ^b^	72.84 ^b^	1.04	<0.01
DM	75.37 ^a^	70.23 ^b^	69.35 ^b^	74.74 ^a^	68.56 ^b^	68.95 ^b^	0.88	<0.01
Ash	14.97 ^a^	18.64 ^a^	2.14 ^b^	24.59 ^a^	18.25 ^a^	19.72 ^a^	4.39	<0.01
OM	78.16 ^a^	72.55 ^b^	74.16 ^b^	77.94 ^a^	71.13 ^b^	73.54 ^b^	0.82	<0.01
EE	80.85 ^a^	86.25 ^a^	57.26 ^b^	95.12 ^a^	94.27 ^a^	94.24 ^a^	5.42	<0.01
TDF	2.71 ^bc^	6.55 ^b^	6.33 ^b^	13.89 ^a^	2.66 ^bc^	2.33 ^c^	2.07	0.02
SDF	5.33 ^c^	9.14 ^c^	12.33 ^c^	38.61 ^b^	48.93 ^a^	10.00 ^c^	3.34	<0.01
IDF	1.58	5.00	4.00	3.67	1.93	1.33	1.22	0.26
NDF	14.03 ^bc^	9.25 ^c^	20.40 ^b^	32.95 ^a^	15.19 ^bc^	9.27 ^c^	3.11	<0.01
ADF	4.50	2.80	7.02	9.04	4.70	1.67	5.46	0.12
Cellulose	5.47	1.82	3.70	8.62	7.02	0.70	8.83	0.39
Hemicellulose	20.38 ^c^	13.15 ^c^	45.48 ^b^	62.96 ^a^	45.35 ^b^	23.27 ^c^	3.45	<0.01

^1^^a,b,c^ Least squares means in a row without a common superscript are different (*P* < 0.05). Each treatment included 6 replicates (*n* = 6). ^2^ ADF = acid detergent fiber; AID = apparent ileal digestibility; CB = corn bran; DE = digestible energy; DM = dry matter; EE = ether extract; IDF = insoluble dietary fiber; NDF = neutral detergent fiber; OB = oat bran; OM = organic matter; RB = rice bran; SDF = soluble dietary fiber; SBP = sugar beet pulp; SH = soybean hulls; TDF = total dietary fiber; WB = wheat bran.

**Table 4:** The apparent total tract digestibilities (ATTDs) of chemical components for pigs fed diets with different high-fiber ingredients ^1^.

	Diets							
Items, % ^2^	WB	CB	SBP	OB	SH	RB	SEM	*P*-Value
DE, MJ/kg	14.11 ^bc^	14.01 ^bc^	15.05 ^a^	14.88 ^a^	14.65 ^ab^	13.70 ^c^	0.12	<0.01
GE	85.03 ^c^	82.50 ^d^	90.27 ^a^	89.38 ^a^	87.14 ^b^	82.51 ^d^	0.66	<0.01
DM	84.66 ^b^	81.59 ^c^	89.82 ^a^	88.49 ^a^	86.72 ^ab^	78.90 ^d^	0.71	<0.01
Ash	28.28 ^b^	43.34 ^a^	41.84 ^a^	41.81 ^a^	51.24 ^a^	26.57 ^b^	2.62	<0.01
OM	87.27 ^b^	83.30 ^c^	92.29 ^a^	91.47 ^a^	88.53 ^b^	83.77 ^c^	0.63	<0.01
EE	60.65 ^a^	57.88 ^a^	18.80 ^c^	67.26 ^a^	40.39 ^b^	40.47 ^b^	3.87	<0.01
TDF	37.78 ^b^	30.31 ^b^	72.54 ^a^	71.87 ^a^	72.32 ^a^	39.13 ^b^	3.52	<0.01
SDF	65.75 ^b^	68.28 ^b^	93.55 ^a^	96.31 ^a^	86.38 ^a^	43.51 ^c^	5.47	<0.01
IDF	37.01 ^d^	28.68 ^de^	60.80 ^ab^	47.72 ^bc^	69.48 ^a^	38.83 ^cd^	4.31	<0.01
NDF	35.91 ^b^	36.46 ^b^	69.59 ^a^	76.27 ^a^	68.59 ^a^	32.77 ^b^	2.83	<0.01
ADF	13.74 ^d^	28.41 ^c^	65.17 ^a^	54.22 ^b^	62.27 ^ab^	12.13 ^d^	3.06	<0.01
Cellulose	14.85 ^d^	32.31 ^c^	62.52 ^a^	51.82 ^b^	64.76 ^a^	31.53 ^c^	3.71	<0.01
Hemicellulose	47.49 ^c^	39.07 ^c^	73.62 ^b^	85.91 ^a^	76.57 ^b^	46.29 ^c^	3.17	<0.01

^1^^a,b,c,d,e^ Least squares means in a row without a common superscript are different (*P* < 0.05). Each treatment included 6 replicates (*n* = 6). ^2^ ADF = acid detergent fiber; AID = apparent ileal digestibility; CB = corn bran; DE = digestible energy; DM = dry matter; EE = ether extract; IDF = insoluble dietary fiber; NDF = neutral detergent fiber; OB = oat bran; OM = organic matter; RB = rice bran; SDF = soluble dietary fiber; SBP = sugar beet pulp; SH = soybean hulls; TDF = total dietary fiber; WB = wheat bran.

**Table 5:** The hindgut disappearance of chemical components in pigs fed diets with different high-fiber ingredients ^1^.

	Diets							
Items, % ^2^	WB	CB	SBP	OB	SH	RB	SEM	*P*-Value
DE, MJ/kg	1.59 ^c^	1.78 ^c^	2.90 ^a^	1.93 ^bc^	2.58 ^ab^	1.61 ^c^	0.02	<0.01
GE	9.60 ^b^	10.46 ^b^	17.38 ^a^	11.61 ^b^	15.27 ^a^	9.67 ^b^	0.22	<0.01
DM	9.29 ^c^	11.36 ^bc^	20.47 ^a^	13.75 ^b^	18.16 ^a^	9.95 ^c^	0.24	<0.01
Ash	13.31 ^cd^	24.70 ^ab^	39.70 ^a^	17.22 ^b^	32.99 ^a^	6.85 ^d^	2.20	<0.01
OM	9.11 ^c^	10.75 ^c^	18.13 ^a^	13.53 ^b^	17.40 ^a^	10.23 ^c^	0.21	<0.01
EE	−20.30	−18.37	−38.46	−27.86	−53.88	−53.77	3.82	0.06
TDF	35.05 ^c^	23.76 ^d^	66.21 ^a^	57.98 ^b^	69.66 ^a^	36.80 ^c^	3.25	<0.01
SDF	30.42 ^c^	59.14 ^b^	81.22 ^a^	57.70 ^b^	37.45 ^c^	33.51 ^c^	3.63	<0.01
IDF	35.43 ^b^	23.68 ^c^	56.80 ^a^	44.05 ^b^	67.55 ^a^	37.50 ^b^	3.05	<0.01
NDF	21.88 ^c^	27.21 ^c^	49.19 ^a^	43.27 ^b^	53.40 ^a^	23.50 ^c^	2.31	<0.01
ADF	10.35 ^d^	25.61 ^c^	58.15 ^a^	45.18 ^b^	57.57 ^a^	10.56 ^d^	2.42	<0.01
Cellulose	9.38 ^d^	30.49 ^c^	58.82 ^a^	43.20 ^b^	57.74 ^a^	30.83 ^c^	2.75	<0.01
Hemicellulose	27.09	25.92	28.14	22.95	31.22	23.02	2.48	0.42

^1^^a,b,c,d^ Least squares means in a row without a common superscript are different (*P* < 0.05). Each treatment included 6 replicates (*n* = 6). ^2^ ADF = acid detergent fiber; AID = apparent ileal digestibility; CB = corn bran; DE = digestible energy; DM = dry matter; EE = ether extract; IDF = insoluble dietary fiber; NDF = neutral detergent fiber; OB = oat bran; OM = organic matter; RB = rice bran; SDF = soluble dietary fiber; SBP = sugar beet pulp; SH = soybean hulls; TDF = total dietary fiber; WB = wheat bran.

**Table 6:** Coefficients of correlation among the AIDs of non-fibrous components or ileal fermentation of fiber components and VFA concentration in ileal digesta of pigs ^1^.

Items ^2^	Acetate	Propionate	Butyrate	Total VFA	GE Digestibility	DM Digestibility	OM Digestibility	CP Digestibility	TDF Digestibility	SDF Digestibility	IDF Digestibility	NDF Digestibility	ADF Digestibility	Cellulose Digestibility	Hemicellulose Digestibility
Acetate	1	0.62	0.40	0.12	−0.82	−0.76	−0.78	−0.42	−0.49	0.16	0.08	−0.36	−0.17	−0.16	−0.03
Propionate		1	0.38	−0.14	−0.63	−0.64	−0.70	0.09	−0.70	0.43	−0.59	−0.46	−0.45	−0.08	−0.06
Butyrate			1	0.20	−0.18	−0.55	−0.49	0.45	0.30	0.76	0.16	0.41	0.37	0.30	0.73
Total VFA				1	0.13	0.24	0.10	0.22	0.59	0.48	0.36	0.55	0.71	0.86 **	0.42
GE digestibility					1	0.89 **	0.92 ***	0.29	0.68	0.14	−0.02	0.77	0.67	0.63	0.48
DM digestibility						1	0.94 ***	0.07	0.45	−0.08	−0.03	0.50	0.46	0.53	0.13
OM digestibility							1	−0.06	0.45	−0.21	−0.09	0.56	0.51	0.42	0.21
CP digestibility								1	0.48	0.66	0.03	0.33	0.12	0.36	0.37
TDF digestibility									1	0.32	0.63	0.85 **	0.77	0.49	0.64
SDF digestibility										1	−0.07	0.52	0.47	0.75	0.75 *
IDF digestibility											1	0.26	0.34	−0.05	0.10
NDF digestibility												1	0.96 ***	0.78	0.90 **
ADF digestibility													1	0.78	0.87 **
Cellulose digestibility														1	0.76
Hemicellulose digestibility															1

^1^ ** Represents 0.05 < *P* < 0.01; *** represents *P* < 0.01. ^2^ ADF = acid detergent fiber; AID = apparent ileal digestibility; CP = crude protein; DM = dry matter; IDF = insoluble dietary fiber; NDF = neutral detergent fiber; OM = organic matter; SDF = soluble dietary fiber; VFA = volatile fatty acid; TDF = total dietary fiber.

**Table 7:** Coefficients of correlation among the ATTDs of non-fibrous components or intact fermentation of fiber components and VFA concentration in feces of pigs ^1^.

Items ^2^	Acetate	Propionate	Butyrate	Total VFA	GE Digestibility	DM Digestibility	OM Digestibility	TDF Digestibility	SDF Digestibility	IDF Digestibility	NDF Digestibility	ADF Digestibility	Cellulose Digestibility	Hemicellulose Digestibility
Acetate	1	0.94 ***	0.99 ***	0.99 ***	0.47	0.53	0.42	0.64	0.55	0.81 **	0.60	0.75	0.77	0.54
Propionate		1	0.94 ***	0.97 ***	0.22	0.32	0.17	0.43	0.39	0.64	0.40	0.57	0.58	0.35
Butyrate			1	0.99 ***	0.53	0.60	0.49	0.64	0.56	0.78	0.64	0.77	0.75	0.60
Total VFA				1	0.42	0.49	0.37	0.58	0.50	0.75	0.58	0.70	0.70	0.51
GE digestibility					1	0.97 ***	0.99 ***	0.81 **	0.78	0.64	0.92 ***	0.85 **	0.73	0.91 ***
DM digestibility						1	0.96 ***	0.72	0.76	0.56	0.89 **	0.85 **	0.67	0.84 **
OM digestibility							1	0.75	0.71	0.57	0.89 **	0.79	0.66	0.88 **
TDF digestibility								1	0.90 **	0.93 ***	0.94 ***	0.93 ***	0.96 ***	0.92 ***
SDF digestibility									1	0.78	0.92 ***	0.95 ***	0.90 **	0.83 **
IDF digestibility										1	0.80	0.87 **	0.97 ***	0.77
NDF digestibility											1	0.94 ***	0.88 **	0.98 ***
ADF digestibility												1	0.95 ***	0.86 **
Cellulose digestibility													1	0.82 **
Hemicellulose digestibility														1

^1^ ** Represents 0.05 < *P* < 0.01; *** represents *P* < 0.01. ^2^ ADF = acid detergent fiber; ATTD = apparent total tract digestibility; DM = dry matter; IDF = insoluble dietary fiber; NDF = neutral detergent fiber; OM = organic matter; SDF = soluble dietary fiber; VFA = volatile fatty acid; TDF = total dietary fiber.

**Table 8:** The prediction equations for fecal VFA concentration by using the ATTDs of different dietary fiber components in pigs ^1^.

Numbers	Equations	R^2^	*P*-Value
Predicted acetate			
1	Acetate (mg/g) = 0.0621 × ATTD of ADF (%) + 5.1927	0.55	0.09
2	Acetate (mg/g) = 0.0976 × ATTD of IDF (%) + 3.2321	0.72	0.03
3	Acetate (mg/g) = 0.2078 × ATTD of ADF (%) − 0.1450 × ATTD of SDF (%) + 5.1929	0.85	0.06
Predicted butyrate			
1	Butyrate (mg/g) = 0.0587 × ATTD of IDF (%) − 0.4960	0.65	0.05
Predicted total VFA			
1	Total VFA (mg/g) = 0.2376 × ATTD of IDF (%) + 2.5864	0.61	0.07

^1^ The prediction equation for fecal propionate concentration was not built by using the ATTDs of different fiber components. ADF = acid detergent fiber; ATTD = apparent total tract digestibility; IDF = insoluble dietary fiber; SDF = soluble dietary fiber; VFA = volatile fatty acid.

## Supplementary Materials

The following are available online at https://www.mdpi.com/2076-2615/10/2/263/s1, Table S1: The dietary formulation in the recovery period of pigs after surgery in the present study.
