# Supplementary material for: Relationship between Dietary Fiber Fermentation and Volatile Fatty Acids’ Concentration in Growing Pigs
Source: Animals (Basel). 2020 Feb 7;10(2):263. doi: 10.3390/ani10020263 (PMC7070776; doi:10.3390/ani10020263)
Supplement: Supplementary file 1 [file animals-10-00263-s001.pdf]

Supplementary Table S1 The dietary formulation in the recovery period of pigs after surgery in the present study

Items	Proportion
Corn	81.29
Soybean meal	14.60
Dicalcium phosphate	0.90
Limestone	0.90
NaCl	0.30
Choline	0.06
Premix†	0.50
L-Histidine	0.03
L-Isoleucine	0.17
L-Lysine-HCl	0.63
DL-Methionine	0.04
L-Phenylalanine	0.08
L-Threonine	0.25
L-Tryptophan	0.08
L-Valine	0.17
Total	100
Analyzed nutritive levels, % as fed basis	
Gross energy, MJ/kg	16.19
Dry matter	85.12
Crude protein	15.62
Ether extract	2.41
Neutral detergent fibre	12.22
Acid detergent fibre	2.11
Ash	4.31
Indispensable amino acids	
Arginine	0.75
Histidine	0.46
Leucine	1.51
Isoleucine	0.62
Lysine	1.18
Methionine	0.32
Phenylalanine	0.88
Threonine	0.79
Tryptophan	0.19
Valine	0.79

†Vitamin-mineral premix supplied the following per kg of diet: vitamin A, 5512 IU; vitamin D_3, 2200 IU; vitamin E, 30 mg; vitamin K_3, 2.2 mg; vitamin B_{12}, 27.6 µg; riboflavin, 4 mg; pantothenic acid, 14 mg; niacin, 30 mg; choline chloride, 400 mg; folic acid, 0.7 mg; thiamine, 1.5 mg; pyridoxine, 3 mg; biotin, 44 µg; Mn (MnO), 40 mg; Fe ($FeSO_4 \cdot H_2O$), 75 mg; Zn (ZnO), 100 mg; Cu ($CuSO_4 \cdot 5H_2O$), 100 mg; I (KI), 0.3 mg; Se (Na_2SeO_3), 0.3 mg.
